# Quantification of Scatter Radiation Doses to the Eyes, Thyroid, and Breasts During Plain Abdominal Computed Tomography Scans With and Without Shielding

**DOI:** 10.7759/cureus.97438

**Published:** 2025-11-21

**Authors:** Kamatchi Krishnamoorthy, Anil K Sakalecha, Venkateswarlu Raavi, R. Mahima Kale, Rahul Kotian, Suchithra T

**Affiliations:** 1 Radiation Oncology, Allied Health Science, Sri Devaraj Urs Academy of Higher Education and Research (Deemed to be University), Kolar, IND; 2 Radiodiagnosis, Sri Devaraj Urs Academy of Higher Education and Research (Deemed to be University), Kolar, IND; 3 Cell Biology and Molecular Genetics, Sri Devaraj Urs Academy of Higher Education and Research (Deemed to be University), Kolar, IND; 4 Imaging Technology, Philips Middle East, Turkey, and Africa (META), Dubai, ARE

**Keywords:** computed tomography, patient’s safety, scatter radiation, shielding, thermoluminescence dosimeter

## Abstract

Purpose: The use of computed tomography (CT) has increased tremendously, and at the same time, it has also raised concerns about potential risks such as cataracts and cancers among patients and occupational workers. The majority of studies reported the entrance surface dose to the eyes, thyroid, and breast within the CT scan field of view, without shielding. In the present study, we aim to measure the scatter doses (outside the scan area) to the eyes, thyroid, and breast during a plain abdominal CT scan with (0.5 mm lead equivalence) and without shielding.

Materials and methods: A cross-sectional analytical study was designed for participants (n = 93; 51 males (55%), 42 females (45%)) and with shielding (n = 93; 52 males (56%), 41 females (44%)) who underwent plain abdominal CT scans. Scatter radiation doses to the eyes, thyroid, and breast were measured using a CaSO₄:Dy Teflon thermoluminescence dosimeter.

Results: The measured mean ± standard deviation value of without and with shielding are as follows: right eye (0.126 ± 0.170 mSv, 0.030 ± 0.035 mSv), left eye (0.112 ± 0.133 mSv, 0.029 ± 0.035 mSv), right thyroid (0.203 ± 0.296 mSv, 0.102 ± 0.089 mSv), left thyroid (0.210 ± 0.282 mSv, 0.112 ± 0.085 mSv), right breast (0.658 ± 0.863 mSv, 0.287 ± 0.218 mSv), and left breast (0.626 ± 0.842 mSv, 0.273 ± 0.199 mSv). Scatter doses to the eyes were reduced by 74-75% (p < 0.0001), the thyroid by 47-50% (p < 0.0001), and the breast by 56% (p < 0.0001) with shielding compared to scatter doses received by these organs without shielding.

Conclusions: The present study indicates that organs outside the CT scan area received scatter radiation. Shielding significantly reduced the doses to the eyes, thyroid, and breast. Hence, the findings support the use of shielding whenever feasible during a CT scan.

## Introduction

Ionizing radiation (IR) has been used for decades in applications such as medicine, industry, power generation, and research. The medical applications of IR are enormous and can be broadly categorized into diagnostic and therapeutic applications for various disease conditions [1]. Among diagnostic applications, computed tomography (CT) has been a highly preferred procedure over the last 50 years: it has evolved from diagnostic to pre-implant assessment and surgical guidance, using specialized software [2]. The number of CT scans performed is constantly increasing; in the United States, 70 million CT scans were performed in 2014, 20 times the number documented in 1980 [3]. The global estimate of CT use during 2009-2018 was 403 million (55/1,000 individuals), which is twice that reported in the 2006 report [4]. In addition, the COVID-19 outbreak has significantly increased CT scan use [5,6] by approximately 200-300% in some centers. While CT scans are used for the diagnosis of various disease conditions [7,8], concerns about low-dose IR and its stochastic effects, such as cataracts and cancers [9,10], have increased globally.

The worldwide average effective dose from natural background radiation to an individual is about 2.4 mSv/year [11]. The effective dose received by individuals during a CT scan ranges from 1 to 14 mSv, which is higher than the natural background radiation [12]. Studies have been conducted to determine the dose range and the biological effects of low-dose, low-dose-rate radiation received during a CT scan. Biological changes, such as alterations in gene expression responsible for cell growth, DNA damage, and apoptosis, have been reported in samples collected from patients who underwent CT scans [13-16]. Entrance surface doses to various radiosensitive organs (eye, thyroid, breast, and gonads) were measured using thermoluminescent dosimeters (TLDs), and the risk of stochastic effects, such as cancer, was also estimated using software. Omer et al. reported equivalent doses to the eye lens and thyroid during brain CT scans, with ranges of 11-429.4 mSv and 1-194 mSv, respectively [17]. Robinson et al. measured scatter radiation to the thyroid gland using TLD during a brain CT scan and found a mean dose of 5.26 mSv. Adejoh et al. measured scatter radiation doses to the breast using TLD during head CT and found that the mean dose ranged from 2.2 to 8.5 mSv. These studies highlighted that the radiation dose to the eyes, thyroid, and breast during CT scans needs to be carefully monitored, given their long-term health effects [18,19].

In recent years, amid growing concern, several authors have explored shielding as a potential protective strategy to reduce patient radiation doses during CT scans. Abuzaid et al. measured scatter radiation doses to the thyroid using TLD with and without shielding (0.5 mm Pb equivalent) during a brain CT scan. They observed that shielding reduced scatter radiation by up to 45% [20]. Zalokar and Mekis measured scatter radiation to the breast using TLD during a head CT scan, with and without a lead shield, and observed that scatter radiation doses were reduced by up to 81% [21]. Ito studied the usefulness of 0.5 mm Pb protective glasses for CT assistants to reduce eye lens dose and found that the scatter dose was reduced by 76.1% [22]. The majority of studies used a shield for only one organ or for the area within the CT scan field of view [23]. However, only a few studies have examined scatter doses to organs outside the scan area and the effectiveness of shielding for the eyes, thyroid, and breast during abdominal CT scans [24,25]. Hence, in the present study, we aim to quantify scatter radiation doses (outside the field area) received by the eyes, thyroid, and breast during a plain abdominal CT scan using a TLD disc with and without (0.5 mm Pb equivalence) shielding. Additionally, the study aims to assess the correlation between measured scatter doses and CT parameters such as kVp and DLP.

## Materials and methods

A cross-sectional analytical study was designed, and ethical clearance was obtained from the Central Ethics Committee of Sri Devaraj Urs Academy of Higher Education and Research (approval number: SDUAHER/KLR/CEC/65/2021-22). The study was carried out in the departments of Allied Health Science and Radiodiagnosis at R.L. Jalappa Hospital and Research Centre, Sri Devaraj Urs Academy of Higher Education and Research, Kolar, Karnataka. The study lasted approximately 24 months.

The shielding was used for all odd-numbered patients recruited in the study, while even-numbered patients were assigned to the unshielded group. Thus, every alternate patient underwent a scan with shielding. Shielding and TLD disc placements were standardized, and the details are described. Shielding and TLD placements were performed by the first author (same person) for all patients and visually verified by the second author.

Study participants

Sample size was estimated at 186 based on the efficacy of breast shielding during CT of the head reported by Zalokar et al. A total of 186 patients undergoing abdominal CT scans were included in the study. Participants were recruited based on the inclusion and exclusion criteria. Participants who underwent plain abdominal CT scans and were aged >18 years were included in the present study. The participants who had undergone cataract surgery, thyroidectomy, mastectomy, or radiotherapy previously were excluded from the study. All the participants were recruited for the study after obtaining written informed consent. The shielding was used for all odd-numbered patients recruited in the study, while even-numbered patients were assigned to the unshielded group. Thus, every alternate patient underwent a scan with shielding. In the group without shielding, there were 51 males and 42 females (n = 93), while in the group with shielding, there were 52 males and 41 females (n = 93).

TLD disc and placement on participants

**Figure 1:**
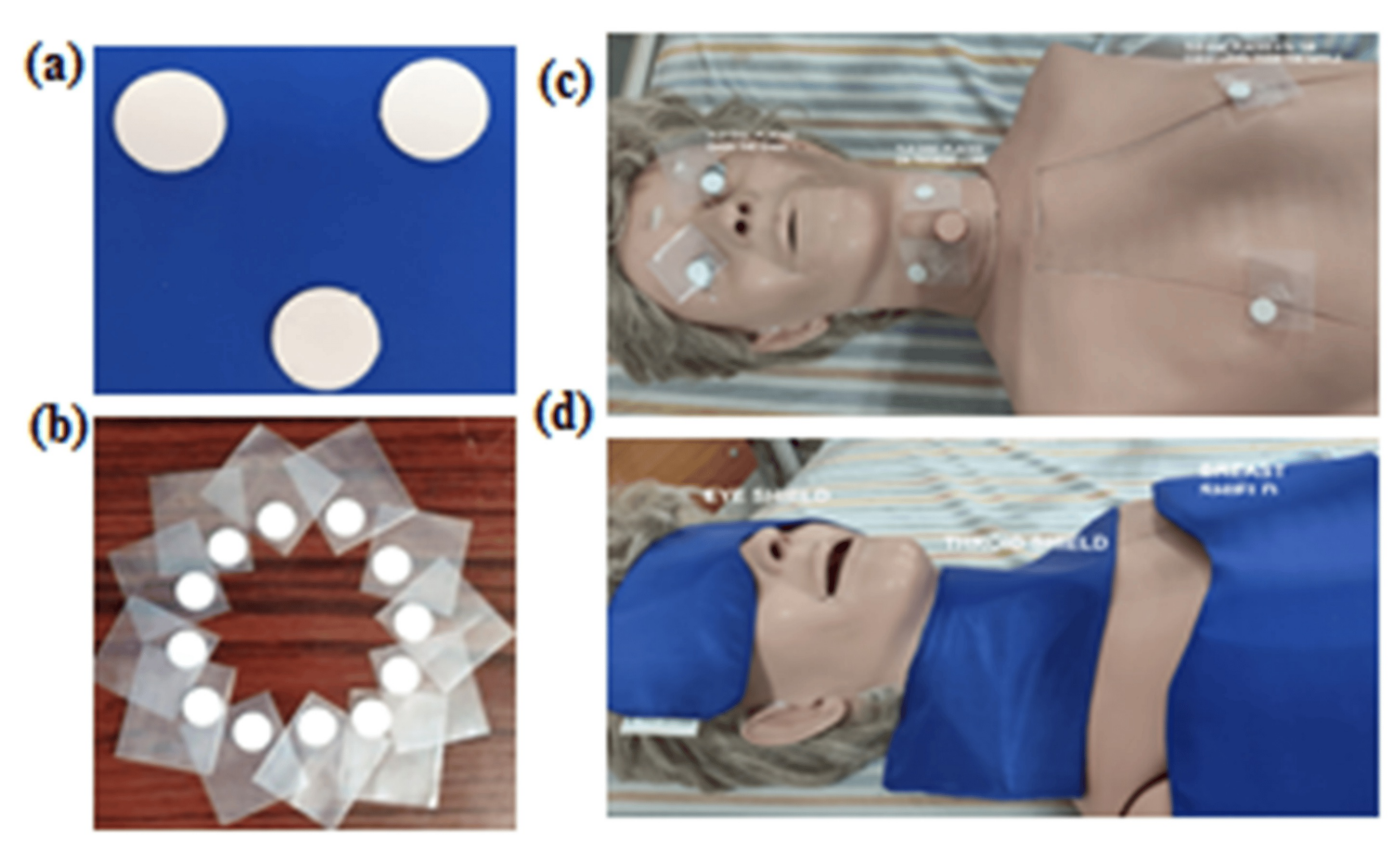
Representative images used in the study (a, b) CaSO₄:Dy Teflon TLD discs. (c) Placement of TLD discs on the eyes, thyroid, and breasts of participants undergoing plain abdominal CT scans. (d) Lead shielding devices are designed for the eyes, thyroid, and breasts. These images demonstrate the methodology for radiation dose measurement and shielding application. TLD: thermoluminescence dosimeter, CT: computed tomography

To measure scatter radiation doses from the CT scan procedure for each study participant, CaSO₄:Dy Teflon discs were used. The representative images of the TLD disc are shown in Figure 1a-1b. TLD disc placements were standardized for all participants. After positioning the patient for a CT scan, the TLD discs were placed over the right eye, the right lobe of the thyroid gland, and the right breast, close to the nipple, and over the left eye, the left lobe of the thyroid gland, and the left breast, close to the nipple. The representative image of the placement of the TLD discs is shown in Figure 1c.

CT scan

All participants underwent an abdominal CT (Somatom Emotion 16-slice spiral CT unit, SIEMENS, USA) scan according to the departmental standard protocol for disease diagnosis. All CT scans were performed without fasting requirements. Patient positioning and scanning procedures were kept consistent for all participants. All CT scans were performed by the same group of trained CT technologists to maintain procedural uniformity.

The CT scan was performed with and without shielding. In the first group, a CT scan was performed without shielding, with TLD discs placed on the eye, thyroid, and breast; in the second group, the CT scan was performed with shielding, with TLD discs placed on the eye, thyroid, and breast. After the CT scan, parameters such as kilovoltage (kVp), milliampere-seconds (mAs), CT dose index (CTDI), and dose-length product (DLP) were collected for each study participant. 

Shielding material

To reduce exposure to scatter radiation during CT scans, we used shielding for radiosensitive organs such as the eye, thyroid, and breast, as reported in previous studies [26-28]. These shields were made up of lead (Pb) with a thickness of 0.5 mm lead equivalent. The representative images of the placement of shielding materials for the eye, thyroid, and breast are shown in Figure 1d. The TLD discs were placed under the shield, and a CT scan was performed in accordance with standard operating procedure. Shielding and TLD placements were performed by the first author (same person) for all patients and visually verified by the second author.

Reading TLD data

The exposed TLD discs from all study participants, after the CT scan (with and without shielding), were carefully packed without exposure to light and sent to an AERB-approved laboratory for analysis. The TLD discs were read using a TLD reader (Intech TLD Reader Model 1602DC/1602PC), and the readings were recorded. The laboratory performed standard TLD calibration, annealing, and background-subtraction procedures in accordance with accredited protocols. Separate unexposed TLD discs were provided for background correction. The TLDs were calibrated for low-energy X-rays consistent with the CT parameters used in this study.

Statistical analysis

The Shapiro-Wilk normality test was performed to determine the data distribution, and normality was confirmed (p > 0.05 for all variables). The obtained data were presented as mean ± standard deviation (SD), with percentages and ranges. An ANOVA was performed to compare the measured scatter doses between the with- and without-shielding groups. A p-value < 0.05 was considered statistically significant. Pearson's correlation analysis was used to assess the association between the registered dose and CT parameters. All calculations and data were analyzed using Excel (Microsoft Corporation, Redmond, WA, USA) and SPSS Statistics version 27 (IBM Corp., Released 2020. IBM SPSS Statistics for Windows, Version 27.0. Armonk, NY: IBM Corp.). The representative graphs were prepared using GraphPad (GraphPad Software Inc., La Jolla, CA, USA).

## Results

Demographic details of the study participants

The study participants (n = 186) were patients who underwent plain abdominal CT scans. The participants were divided into two groups: (i) without shielding (n = 93) and (ii) with shielding (n = 93). The mean age ± SD (range) of the participants without and with shielding is 49.86 ± 12.29 (28-73) and 46.59 ± 12.92 (26-75), respectively. The number of males and females is 51 (55%) and 42 (45%) without shielding and 52 (56%) and 41 (44%) with shielding. The demographic details of study participants are shown in Table 1.

**Table 1:** Demographic details of the study participants who underwent plain abdominal CT scans without and with shielding y: years, SD: standard deviation, CT: computed tomography

Parameter	Without shielding (n = 93)	With shielding (n = 93)
Age (years, mean ± SD)	49.86 ± 12.29	46.59 ± 12.92
Gender		
Male	51 (55%)	52 (56%)
Female	42 (45%)	41 (44%)

CT parameters of the study participants

All study participants underwent an abdominal CT scan to diagnose their condition. The CT scan was performed for both groups using the automatic exposure control technique, which remained consistent across both conditions. The CT parameters, such as kVp, mAs, CTDI, and DLP, were collected for both without and with shielding. The mean ± SD (range) of kVp of the CT scan without and with shielding were 110.86 ± 4.08 (110-130 kVp) and 110.86 ± 3.50 (110-130 kVp), respectively. The mean ± SD (range) of mAs of the CT scan without and with shielding were 74.59 ± 16.35 (39-99) and 73.24 ± 14.90 (37-99), respectively. The mean ± SD (range) CTDI of the CT scan without and with shielding were 4.80 ± 1.49 (2.45-7.96) and 4.77 ± 1.42 (2.54-7.85), respectively. The mean ± SD (range) DLP of the CT scan without and with shielding were 345.29 ± 143.03 (119.74-745.23) and 345.11 ± 131.93 (119.19-697.64), respectively. The mean kVp, mAs, CTDI, and DLP obtained with and without shielding did not differ significantly (p > 0.05). The CT parameters for the study participants are shown in Table 2.

**Table 2:** CT parameters of the study participants who underwent plain abdominal CT scans without and with shielding CT imaging parameters of study participants undergoing plain abdominal CT scans with and without shielding. Data are expressed as mean ± SD. Statistical comparisons were performed using ANOVA; p-values indicate the significance level between groups. The table suggests the shielding's efficiency in reducing radiation dose. The purpose of the table is to quantify the shielding's protective effect. The data are presented as mean ± SD, showing a significant reduction in the scatter dose from 47% to 75%. The p-value remains <0.0001 throughout. The table shows that each column represents the mean dose, the percentage reduction, and the statistical significance. kVp: kilovoltage peak, mAs: milliampere-seconds, CTDI: computed tomography dose index, DLP: dose-length product, mGy: milligray, mGy·cm: milligray-centimeter, SD: standard deviation, n: number of participants, CT: computed tomography, ANOVA: analysis of variance

Parameter	Without shielding (n = 93) mean ± SD	With shielding (n = 93) mean ± SD	p-value
kVp	110.86 ± 4.08	110.86 ± 3.50	0.99
mAs	74.59 ± 16.35	73.24 ± 14.90	0.56
CTDI (mGy)	4.80 ± 1.49	4.77 ± 1.42	0.89
DLP (mGy*cm)	345.29 ± 143.03	345.11 ± 131.93	0.99

Comparison of scatter radiation doses to the eyes, thyroid, and breast during plain abdominal CT scans without and with shielding

**Figure 2:**
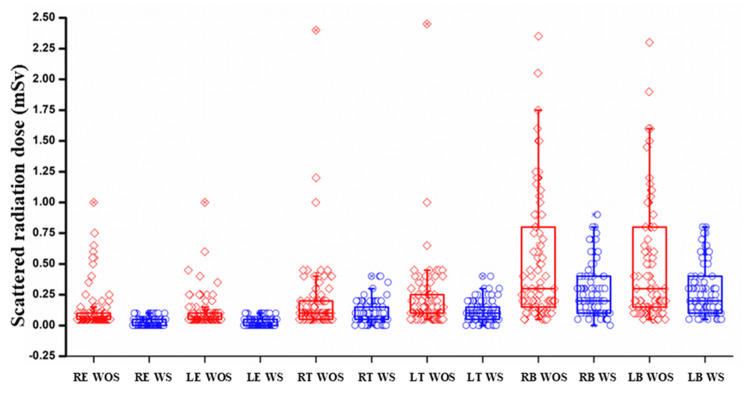
Comparison of scatter radiation doses to the eyes, thyroid, and breast during plain abdominal CT scans without and with shielding The red diamonds represent the radiation dose measurements and are called the WS. The data points mainly show a reduction in radiation levels after using the protection. And the blue circles (WOS), i.e., without a shield, show the data points, mainly the baseline radiation level without adequate protection. This pattern of blue circles and red diamonds shows that the blue circles are higher than the red diamonds, highlighting that the protection has maintained the dose reduction. This visualization shows the protective magnitude and the variation in dose reduction across different anatomical sites. These data points are generally higher, reflecting greater scatter radiation exposure when no protective shielding is used. Blue circles indicate measurements WS. The box plots summarize these distributions, with the diamonds and circles showing the individual values for each subject/measurement. Regarding displaying significance levels on box plots, the conventional approach is to connect paired conditions (e.g., RE WOS vs. RE WS) and label the line with the p-value (p = 0.0001), as in a paired-comparison plot. This visual indicates that the reduction in scatter radiation from shielding is statistically significant for each organ. These points are lower, showing the effectiveness of shielding in reducing radiation dose. RE WOS: right eye without shielding, RE WS: right eye with shielding, LE WOS: left eye without shielding, LE WS: left eye with shielding, RT WOS: right thyroid without shielding, RT WS: right thyroid with shielding, LT WOS: left thyroid without shielding, LT WS: left thyroid with shielding, RB WOS: right breast without shielding, RB WS: right breast with shielding, LB WOS: left breast without shielding, and LB WS: left breast with shielding, CT: computed tomography

To determine which organ received higher or lower scatter doses, the scatter doses were compared. The scatter dose to the breast was significantly higher than to the thyroid and eyes (p < 0.001). Further, the thyroid received higher doses than the eyes. The scatter dose received by the eye, thyroid, and breast is in the following order: breast > thyroid > eyes. The comparison of scatter radiation doses to the eyes, thyroid, and breast, with and without shielding, is shown in Figure 2.

Reduction levels of scatter doses to the eyes, thyroid, and breast during plain abdominal CT scans with shielding

Table 3 shows the effectiveness of shielding in reducing scatter radiation exposure to radiosensitive organs. The data show that both eyes benefited most, with right- and left-eye doses decreasing by 75% and 74%, respectively, from 0.126 ± 0.170 mSv to 0.031 ± 0.035 mSv and from 0.112 ± 0.133 mSv to 0.029 ± 0.035 mSv. The thyroid experienced moderate reductions of 50% on the right and 47% on the left, while the breasts saw a 56% decrease on both sides, with right breast exposure reducing from 0.658 ± 0.863 mSv to 0.287 ± 0.218 mSv and left breast from 0.626 ± 0.842 mSv to 0.273 ± 0.199 mSv. All reductions were statistically significant (p = 0.0001), confirming that shielding substantially lowers scatter radiation doses to critical radiosensitive organs.

**Table 3:** Scattering of the radiation dose for the selected organs in with or without shielding Scatter radiation doses received by radiosensitive organs (eyes, thyroid, and breast) during abdominal CT scans with and without shielding. Data are expressed as mean ± SD. P-values indicate statistical significance between groups. The table assessed the efficiency of the radiopaque shielding by comparing radiation doses to the bilateral radiosensitive organs at the time of image acquisition. Radiation exposure is represented as the mean dose in mSv ± SD for the unshielded and shielded states. In addition, the data show a reduction in radiation across all anatomical locations, with efficacy of 47-75%; the dose has been reduced. The p < 0.0001 is maintained for the statistical significance by the independent samples t-test, which validates the clinical significance of the protection for the organs like the ocular, thyroid, and breast tissue. Rt: right, Lt: left, SD: standard deviation, mSv: millisievert

S. No	Radiosensitive organs	Without shielding mSv	With shielding mSv	Reduction percentage of scatter radiation dose	p-value
1	Rt. Eye	0.126 ± 0.170	0.031 ± 0.035	75%	0.0001
2	Lt. Eye	0.112 ± 0.133	0.029 ± 0.035	74%	0.0001
3	Rt. Thyroid	0.203 ± 0.296	0.102 ± 0.089	50%	0.0001
4	Lt. Thyroid	0.210 ± 0.282	0.112 ± 0.085	47%	0.0001
5	Rt. Breast	0.658 ± 0.863	0.287 ± 0.218	56%	0.0001
6	Lt. Breast	0.626 ± 0.842	0.273 ± 0.199	56%	0.0001

Scatter doses received by the male chest versus the female breast

A subgroup analysis was conducted to examine differences in scatter doses received by the male chest and the female breast among participants in the study with and without shielding. In the shielding group, the mean dose to the right breast among female participants (0.262 ± 0.192 mSv) is lower than that to the right chest among male participants (0.321 ± 0.249 mSv). However, the p-value shows no significant difference between genders (p = 0.214). In the shielding group, the mean dose to the left breast among female participants (0.256 ± 0.184 mSv) is lower than the mean chest dose among male participants (0.302 ± 0.222 mSv). Still, the p-value again shows no significant difference between genders (p = 0.2882).

In the group without shielding, the mean dose to the right breast among female participants (0.637 ± 0.926 mSv) is lower than that to the right chest among male participants (0.675 ± 0.817 mSv). However, the p-value shows no significant difference between genders (p = 0.834). In the group without shielding, the mean dose to the left breast among female participants (0.636 ± 0.895 mSv) is higher than the mean chest dose among male participants (0.620 ± 0.805 mSv). However, the p-value shows no significant difference between genders (p = 0.9279). These findings suggest that there was no significant difference in the scatter doses received by the breast in female participants and by the chest in male participants.

Correlation between scatter doses to the eyes, thyroid, and breast with DLP during plain abdominal CT scans without and with shielding

**Figure 3:**
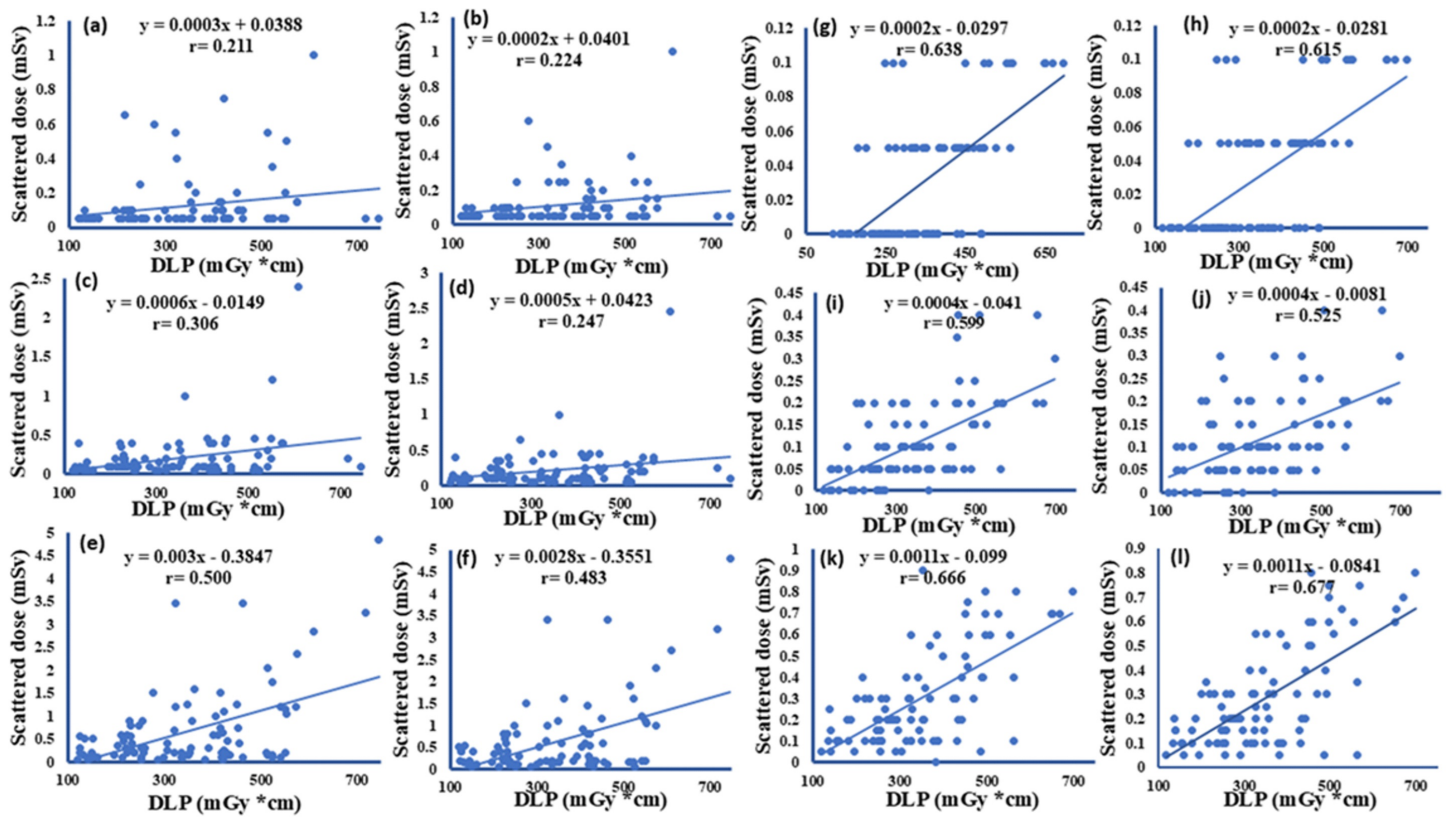
Correlation between DLP and scatter radiation doses to the eyes, thyroid, and breast without (a-f) and with (g-l) shielding during plain abdominal CT scan Without shielding: (a) right eye, (b) left eye, (c) right thyroid, (d) left thyroid, (e) right breast, and (f) left breast. With shielding: (g) right eye, (h) left eye, (i) right thyroid, (j) left thyroid, (k) right breast, and (l) left breast. DLP: dose-length product

To investigate the relationship between scatter doses measured at the eyes, thyroid, and breast and the DLP, a correlation analysis was performed. Without shielding, weak to moderate positive correlations were observed. Specifically, the right eye showed a correlation of r = 0.21 (p = 0.034), and the left eye showed a correlation of r = 0.22 (p = 0.029) with DLP. The right thyroid demonstrated r = 0.31 (p = 0.008), and the left thyroid r = 0.25 (p = 0.015). For the breasts, the correlations were stronger, with the right breast showing r = 0.50 (p < 0.001) and the left breast r = 0.48 (p < 0.001). These results indicate that, without shielding, higher DLP values are associated with higher scatter doses, particularly at the breast. With shielding, the correlations between scatter doses and DLP became notably stronger. The right eye showed r = 0.64 (p < 0.001), and the left eye showed r = 0.62 (p < 0.001). For the thyroid, the right side had r = 0.60 (p < 0.001) and the left side had r = 0.53 (p < 0.001). The breast demonstrated the highest correlations, with r = 0.67 (p < 0.001) for the right breast and r = 0.68 (p < 0.001) for the left breast. These findings suggest that shielding not only reduces scatter radiation. With shielding, the relationship between DLP and scatter dose remained positive but less variable. All correlation coefficients are statistically significant (p < 0.05), and the p-value is < 0.001 for all measured organs (Figure 3).

## Discussion

CT imaging is a foremost diagnostic tool in medicine, used to diagnose various disease conditions [13]. Despite the advantages of this modality, the low-dose and dose-rate radiation received by the patients during CT is a significant concern due to long-term health effects [8-10,29]. The management of low-dose radiation in CT is a complex task that requires advanced dose reduction strategies, appropriate protocols, and optimization of imaging parameters [14]. CT machine manufacturers and healthcare professionals continuously work to reduce patient radiation dose without compromising scan quality and to improve the technology and efficiency of diagnosis [30]. It is important to note that CT scans are used for early disease detection and diagnosis; the benefits often outweigh the risk of exposure to low-dose radiation. Health professionals carefully evaluate the risk-to-benefit ratio before recommending a CT scan and ensuring that the diagnostic value justifies the radiation exposure [1]. To determine the range of entrance surface/scatter doses, several studies have used TLD materials/software tools at radiosensitive organs, such as the eyes, thyroid, and breast, during various CT scans [18-21]. Few studies have also reported the use of shielding to reduce the entrance surface dose and scatter radiation dose to patients during CT scans. Those reported studies mainly focused on doses within the field area, used shielding for either one or two organs, and used phantom models.

The CT parameters, such as kVp, mAs, CTDI, and DLP values, obtained with and without shielding groups did not differ significantly (Table 2) because the CT scan parameters were kept consistent across both groups. To find the range of scatter radiation doses received by the radiosensitive organs, eyes, thyroid, and breast (outside the scan area), we measured the doses using the TLD material (CaSO₄:Dy Teflon discs). Scatter radiation doses to the eyes without shielding during a plain abdominal CT scan in the present study range between 0.05 and 1 mSv. This indicates that the eyes are exposed to a certain amount of scatter radiation during plain abdominal CT scans. The results of this study are consistent with those of Ito et al., who measured scatter doses to the eyes (0.422 mGy) in CT assistants without any protective device [22].

Similarly, another study reported the absorbed doses (0.08 mGy) to the eyes during a CT chest scan on a phantom using TLD. The scatter radiation doses to the thyroid without shielding during a plain abdominal CT scan in the present study range between 0.05 and 2.45 mSv. These results indicate that the thyroid also received a significant amount of scatter radiation doses during a plain abdominal CT scan. The results obtained in this study are within the range of scatter thyroid doses reported by Robinson et al. (3.433 ± 2.787 mSv) during brain CT scans [18].

Similarly, the study by Abuzaid et al. found that the thyroid radiation dose ranged from 0.13 to 0.26 mSv. The range of scatter radiation doses to the breast without shielding during a plain abdominal CT scan in the present study is 0.05-4.85 mSv. The results indicate that, as with the eyes and thyroid, the breast also received significant scatter radiation during a plain abdominal CT scan [20]. Scatter doses to the breast reported in this study were similar to those reported by Zalokar and Mekis, who measured the skin dose (0.338 ± 0.04 mSv) to the breast during head CT scans using TLD material [21].

Similarly, Adejoh et al. reported scatter radiation doses (3.74 ± 2.28 mGy) to the breast during a head CT scan using a phantom and TLD material [19]. These results indicate that the plain abdominal CT scan delivered scatter radiation to radiation-sensitive organs such as the eyes, thyroid, and breast. The increase in registered scatter radiation doses follows the order: breast > thyroid > eyes. This indicates that the breast, which is close to the CT scan field, received a higher scatter radiation dose, and the eyes, which are far from the CT scan field, received a lower scatter radiation dose during plain abdominal CT scans. Several studies have reported that low-dose radiation can increase DNA damage [15], as measured by dicentric assay, micronucleus assay, γ-H2AX assay, gene expression assays, etc. [16], and can have long-term health effects [30].

The CT scan without shielding registered significant scatter radiation doses to the eye, thyroid, and breast. Further, to determine the range of scatter doses with shielding, we used 0.5 mm Pb-equivalent shielding material and measured scatter radiation doses by placing TLD discs beneath the shields. Scatter radiation doses to the eyes with shielding in the present study range between 0 and 0.1 mSv. Scatter radiation doses to the right and left eyes were reduced by 75% and 74%, respectively, with shielding. In other words, the maximum dose registered in the eyes with shielding was reduced to 0.1 mSv compared to 1 mSv without shielding (Table 3). Scatter radiation doses to the thyroid with shielding in the present study range between 0 and 0.4 mSv. Scatter radiation doses to the right and left thyroid were reduced by 50% and 47%, respectively, with shielding. In other words, the maximum thyroid dose with shielding was reduced to 0.4 mSv, compared with 2.45 mSv without shielding (Table 3).

Abuzaid et al. observed that scatter radiation doses to the thyroid were reduced by 45% when 0.5 mm Pb-equivalent shielding was used during a brain CT scan [20]. Scatter radiation doses to the breast with shielding in the present study range between 0 and 0.9 mSv. Scatter radiation doses to the right and left breasts were reduced by around 56% with shielding. In other words, the maximum dose registered at the breast with shielding was reduced to 0.9 mSv compared to 4.85 mSv without shielding (Table 3). Zalokar and Mekis reported that scatter radiation doses to the breast were reduced by 81% with 0.5 mm Pb-equivalent shielding during a head CT scan [21]. The order of the reduction of scatter radiation doses was as follows: eyes > breast > thyroid. The results indicate that Pb shielding significantly reduced scatter radiation doses to radiation-sensitive organs, such as the eyes, thyroid, and breast.

Since we included both male and female participants, we performed a subgroup analysis to examine differences in scatter doses to the breast in female patients vs. the chest in male subjects. We found that scatter doses did not differ significantly. Furthermore, to assess the association between registered scatter radiation doses and DLP, a correlation analysis was performed. The DLP vs. eyes (right: r = 0.21, r = 0.64, left: r = 0.22, and r = 0.62), thyroid (right: r = 0.31, r = 0.60, left: r = 0.25, and r = 0.53), and breast (right: r = 0.50, r = 0.67, left: r = 0.48, and r = 0.68) for without and with shielding. The registered scatter doses were weakly correlated without shielding and moderately correlated with shielding. The lack of correlation might be due to the DLP being the dose from the scan area, whereas the measured dose was the scatter radiation dose outside the CT scan field. Overall, monitoring the patient's radiation dose during a CT scan is essential to reduce long-term health effects.

Non-random allocation of shielding between patients is a limitation of the study. It may have introduced selection bias, potentially affecting the generalizability of the results. Future studies employing randomized allocation are recommended to minimize such bias.

Recent international guidelines from the International Commission on Radiological Protection and the American Association of Physicists in Medicine do not support routine patient shielding in CT examinations for two reasons. First, modern dose-optimization technologies (e.g., automatic exposure control and iterative reconstruction) already minimize unnecessary exposure, and second, shielding may interfere with these systems or degrade image quality. However, the present study and its findings remain relevant, as they explore the selective use of smaller lead shielding for radiosensitive organs outside the scanned area. Hence, the chances of shielding interfering with automatic exposure control systems or degrading the image quality are minimal. Though automatic exposure control and iterative reconstruction technologies can reduce unnecessary exposure, the recent trend toward increased CT use leads to cumulative long-term dose exposure, especially to radiosensitive organs, which can result in stochastic effects such as radiation-induced malignancies. Hence, exploring ways to reduce scatter doses to radiosensitive organs remains relevant even in the era of dose-optimization technologies. In line with this, the present study’s purpose is not to advocate for shielding use but to quantify and compare scatter radiation levels with and without shielding for research and dosimetric understanding.

## Conclusions

The present study results indicate that a significant amount of scatter radiation doses is recorded in radiosensitive organs, such as the eyes, thyroid, and breast (outside the CT scan area), in patients undergoing plain abdominal CT scans. The use of 0.5 mm Pb equivalent shielding significantly reduced scatter radiation doses to the eyes, thyroid, and breast of patients compared with patients without shielding. Measuring scatter radiation doses and using shielding can help monitor and reduce low-dose radiation exposure to radiosensitive organs during CT scans, thereby lowering the risk of long-term health effects.
